# Immunodominant IgM Epitopes of the *Angiostrongylus cantonensis* Galectin-1 and Galectin-2 Proteins Recognized by Patients’ Sera: Optimization of an ELISA Assay for Human Acute Diagnosis of Angiostrongyliasis

**DOI:** 10.3390/ijms27125381

**Published:** 2026-06-15

**Authors:** Paloma Napoleão-Pêgo, Guilherme C. Lechuga, João P. R. S. Carvalho, Flávio R. da Silva, Karyne Rangel, Mariana S. Freita, Jessica A. Waterman, Arnaldo Mandonado-Junior, Carlos Graeff-Teixeira, Salvatore G. De-Simone

**Affiliations:** 1 Center for Technological Development in Health (CDTS), Oswaldo Cruz Foundation (FIOCRUZ), Rio de Janeiro 22040-036, RJ, Brazil; pegopn@fiocruz.br (P.N.-P.); guilherme.curty@fiocruz.br (G.C.L.); joao.rangel@fiocruz.br (J.P.R.S.C.); flavio.rocha@fiocruz.br (F.R.d.S.); karyne.rangelk@gmail.com (K.R.); mariana.freitas@fiocruz.br (M.S.F.); jessicawaterman@aluno.fiocruz.br (J.A.W.); 2 Post-Graduation Program on Science and Biotechnology, Federal Fluminense University, Niterói 22040-036, RJ, Brazil; 3 Program of Post-Graduation on Parasitic Biology, Oswaldo Cruz Institute, Oswaldo Cruz Foundation, Rio de Janeiro 21040-900, RJ, Brazil; 4 Laboratory of Biology and Parasitology of Wild Mammals Reservoirs, Oswaldo Cruz Institute, Oswaldo Cruz Foundation, Rio de Janeiro 21040-360, RJ, Brazil; maldonado@ioc.fiocruz.br; 5 Infectious Diseases Unit, Department of Pathology, Federal University of Espirito Santo, Vitória 29075-910, ES, Brazil; carlos.teixeira@ufes.br

**Keywords:** *Angiostrongylus cantonensis*, galectin-1, galectin-2, IgM epitopes, immunological diagnosis, chimeric polypeptides, ELISA

## Abstract

Angiostrongyliasis, the primary cause of eosinophilic meningitis, represents an emerging disease caused by *Angiostrongylus cantonensis* larvae, inadvertently transmitted to humans. The diagnosis of human angiostrongyliasis relies on epidemiological features, clinical symptoms, medical history, and laboratory findings, notably hyper eosinophilia in blood and cerebrospinal fluid. Consequently, accurate diagnosis is challenging and prone to confusion with other parasitic diseases. The quest for an early, rapid, and specific diagnostic test for angiostrongyliasis persists, driven by the imperative for enhanced test specificity. This study focused on mapping IgM epitopes on galectin-1 (Gal-1) and galectin-2 (Gal-2) proteins derived from *A. cantonensis.* The specificity of the epitopes was assessed using database homology analysis. After selecting specific epitopes, researchers chemically synthesized 12 individual multi-antigen peptides (MAPs4) and one chimeric polypeptide that is 65 amino acids long. The effectiveness of these synthesized peptides was subsequently evaluated using enzyme-linked immunoassay (ELISA). A total of twelve unique IgM epitopes were discovered; five were linked to Gal-1, while seven were linked to Gal-2. An ELISA-peptide method confirmed the twelve epitopes, and then the chimeric polypeptide was employed as an antigen to coat ELISA plates. This setup was evaluated with patients’ sera to diagnose strongyloidiasis in vitro. This study provides a comprehensive representation of the IgM epitopes of Gal-1 and Gal-2 from *A. cantonensis*. ELISA data utilizing the chimeric polypeptide demonstrate that the selected sequences hold promise for the development of a specific immunological assay tailored for the acute diagnosis of angiostrongyliasis infections.

## 1. Introduction

Angiostrongyliasis is an important foodborne zoonosis caused by infection with the nematode species *Angiostrongylus costaricensis* and *Angiostrongylus cantonensis*. This parasite primarily infects rodents, particularly rats, but can also infect other mammals, including humans.

These two species are most commonly found in tropical and subtropical regions and have a significant public health impact in various parts of the world, including the Americas (South America and the Caribbean), Asia, the Pacific basin, Europe, India, and China [1,2,3].

In both cases, rats serve as the primary definitive hosts, and snails function as intermediate hosts [4,5]. The spread of these organisms is often linked to the movement of their primary hosts (rats) and intermediate hosts (snails), and climate change may also influence their distribution [1,5,6].

Thousands of human cases have been recorded worldwide so far [7,8]. Human infection primarily occurs through the consumption of undercooked snails infected with parasites (Pila or Pomacea snails) [8] or through the consumption of vegetables contaminated with infective third-stage larvae (L3) [9,10,11].

Symptoms of angiostrongyliasis can vary with the severity of the infection but commonly include headache, neck stiffness, nausea, vomiting, fever, fatigue, and sensory disturbances. In more severe cases, the infection can lead to meningitis, encephalitis, paralysis, and even death.

The treatment for angiostrongyliasis generally involves supportive care to manage symptoms, as no specific antiparasitic drugs are currently approved for human use [12]. For serious conditions, corticosteroids can help lower inflammation within the central nervous system (CNS). Although albendazole is sometimes used in combination with corticosteroids, there are ongoing concerns that anti-helminthic drugs might worsen symptoms by triggering a systemic reaction when the worms die [13]. A lumbar puncture is required to identify the cause of eosinophilic meningitis and, if necessary, can be repeated to help reduce intracranial pressure [14].

Prevention of angiostrongyliasis primarily involves avoiding the consumption of raw or undercooked intermediate hosts, practicing good food hygiene, thoroughly washing fruits and vegetables, and avoiding contact with potentially contaminated environments, such as areas with rat populations.

The diagnosis of angiostrongyliasis is challenging because its symptoms may mimic those of other conditions, such as paragonimiasis, gnathostomiasis, and cysticercosis [15].

In addition, diagnosis is typically presumptive, based on clinical and epidemiologic criteria in people with otherwise unexplained eosinophilic meningitis. Laboratory tests, such as cerebrospinal fluid analysis, can aid in diagnosis. Imaging studies, such as MRI or CT scans, may also reveal characteristic changes in the brain or spinal cord, but direct detection of the parasite in patients is rare [16,17,18]. The sensitivity of parasitological and molecular methods is unsatisfactory for diagnosing strongyloidiasis, and serological techniques remain the most effective diagnostic approach.

Immunodiagnostic tests have been developed in research settings but are still not approved or licensed for clinical use. IgG antibody-based tests are the primary technology used for clinical measurements of diagnostic markers. Over the past decades, several immunological tests have been developed to support clinical diagnosis, using crude somatic antigens or partially purified antigens from *A. cantonensis* adult worms [19], brain-stage larvae, excretory-secretory products, and recombinant proteins [20,21].

Antigenic proteins with molecular masses of 29–31 kDa from *A. cantonensis* worms have been used as primary markers in immunological diagnosis of angiostrongyliasis [22,23,24], but cross-reactivity with other parasites has been reported [25,26,27]. Other studies identified a 31 kDa *A. cantonensis* antigen as galectin-2 (*Acan*-Gal-2) [28], and a recombinant *A. cantonensis* galectin-2 (rAcGal2) protein was produced [28] to develop an immunochromatographic test (ICT) [29,30] for diagnosing human angiostrongyliasis. However, these assays have not been evaluated for potential cross-reactivity or their diagnostic accuracy during the early stage of infection. Conversely, galectin-1 has emerged as a critical molecule at the intersection of host–pathogen interaction [31], immune regulation [32,33], and diagnostic/therapeutic relevance in other pathogens [34].

Therefore, this study reports the complete IgM epitope mapping of *A. cantonensis* (*Acan*) galectin-1 (*Acan-*Gal-1) and *Acan-*Gal-2 to identify specific epitopes and to develop an IgM-ELISA-peptide assay with high specificity and sensitivity for the early diagnosis of infection.

## 2. Results

### 2.1. Identification and Mapping of Linear Epitopes Using Synthetic Peptides

**Figure 1:**
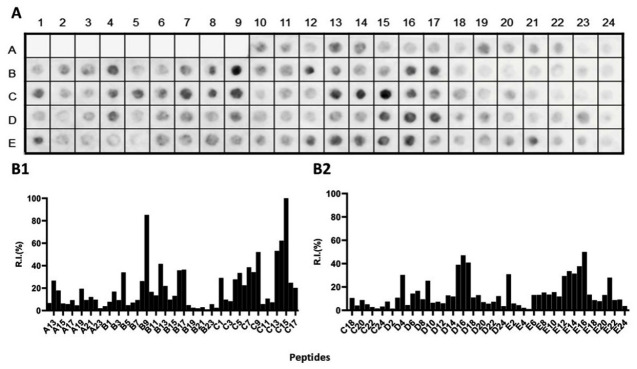
IgM epitopes mapping of *Acan-*Gal-1 (285 aa; AEK98124.1) and *Acan-*Gal-2 (278 aa; AEK98123.1) proteins from *A. cantonensis* using the SPOT synthesis array. (**A**) A set of peptides, every 15 amino acids long and overlapping by 10 residues, was tested with serum samples (*n* = 7) from patients. Panel A highlights the peptides that react with IgM, whereas panels (**B1**,**B2**) from patients. Panel A highlights the peptides that react with IgM, whereas panels (**B1**,**B2**) display the signal intensities (SI). A1–A9 negative controls, A10 (GYPKDGNAFNNLDRI-*C. tetani*) and A11 (KEVPALTAVE TGATN–Poliovirus) positive controls. Tables S1 and S2 provide details on the peptides that match the coding regions of the *Acan-*Gal-2 (A12–C17) and Gal-1 (C18–E24) proteins. The position number on the Spot-synthesis membrane identifies each peptide. Any spot intensities under 30% were treated as background.

The epitopes of the *Acan-*Gal-1 and *Acan-*Gal-2 from *A. cantonensis* recognized by patients’ sera were mapped using the parallel Spot-synthesis strategy. The peptide library consisted of 2 × 33 peptide sequences, each 15 amino acids long, overlapping by 10 amino acids, and covering the entire protein sequence. A representative experiment is presented in Figure 1. The list of synthesized peptides is shown in Table S1. Overall, each epitope displayed relatively strong reactivity (with a 9–15-amino-acid extension). However, the highest intensity was observed with the antigenic determinants in peptides B9 (**IFVDQKELKEYEHRL**) and C15 (**LEPHELNGLQIGGDV**) from Gal-2 (80–100%). The most reactive peptides from *Aca*n-Gal-1 were D16 (RFQVVIDQKEFRDYE) and E16 (EKGVGFDLAIKNESY), but the intensity was lower (50%) than the peptides from *Acan-*Gal-2, indicating that *Acan-*Gal-2 is more immunogenic than *Acan-*Gal-1.

Analysis of spot signal intensity for the synthesized peptides derived from the *Acan*-Gal-1 and *Acan*-Gal-2 sequences revealed 4 epitopes for Gal-1 and 8 for Gal-2 (Table 1). The secondary structure of the identified epitopes is also presented in Table 1 and was based on the I-Tasser predictions (accessed on 10 March 2025).

### 2.2. Spatial Location of the Most Reactive Acan-Gal-1 and Acan-Gal-2 Epitopes

**Figure 2:**
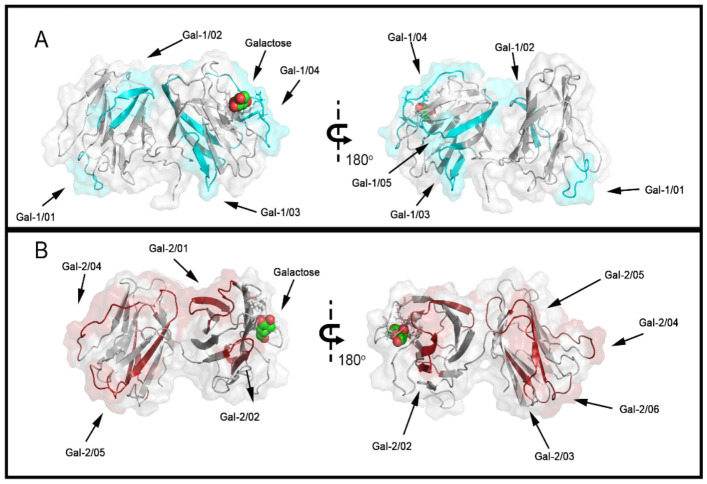
Three-dimensional structures of *Acan-*Gal-1 (**A**) and *Acan-*Gal-2 (**B**), showing the locations of epitopes recognized by human anti-Angiostrongylus IgM antibodies. Epitopes in *Acan-*Gal-1 were highlighted in blue (**A**), and those in *Acan*-Gal-2 in red (**B**), indicating a diverse surface pattern that may bind polyclonal IgM antibodies. Galactose molecules (red/green) and the galactose binding site was highlighted in the protein structure. Three-dimensional molecular structure of the parasite *Aca*-Gal-1 (AF-G1EUS1-F1-v4) and *Acan*-Gal-2 (AF-G1EUS2-F1-v4) was retrieved from AlphaFold.

To analyze the spatial localization of the identified antigenic determinants, three-dimensional (3D) structural models of each target protein were generated using the I-TASSER server (Figure 2A,B). This structure-based approach enabled the mapping of experimentally identified linear epitopes onto the proteins’ predicted tertiary structures via SPOT synthesis. In total, five epitopes in *Acan*-Gal-1 and seven in *Acan*-Gal-2 (Table 1) were projected onto the structural models, enabling evaluation of their topological distribution and potential accessibility to antibody recognition.

Structural mapping revealed that the majority of the twelve epitopes were located within loop or coil regions of the proteins. These structural motifs are typically characterized by higher conformational flexibility and a lack of stable secondary structure compared with α-helices or β-sheets. Because loop regions frequently protrude from the protein core and are less constrained by intramolecular hydrogen bonding, they tend to be more exposed to the surrounding solvent environment. Such solvent exposure is a critical feature of immunodominant regions, as it facilitates interaction with antibodies and B-cell receptors.

The hydropathy plots of both proteins also suggested that residues in all epitopes are predominantly located in hydrophilic segments of the sequence.

### 2.3. Epitopes and Glycosylation

The full-length Acan-Gal-1 protein is composed of 285 amino acids and features a tandem-repeat architecture consisting of an N-terminal carbohydrate recognition domain (NCRD, residues 1–150) and a C-terminal CRD (CCRD, residues 158–285), connected by a short linker region (residues 151–157). Conserved motifs associated with carbohydrate-binding activity are present in both the NCRD and CCRD [35,36].

To determine whether glycosylation might hinder or interfere with antibody binding to key epitopes, we evaluated the presence of predicted glycosylation sites within the identified epitope sequences. As shown in Table 1, only four epitopes [Gal-1/01 (N-linked), Gal-1/03 (both N- and O-linked), and Gal-1/04 (WGXEER motif)] are predicted to be susceptible to glycosylation. The *Acan*-Gal-2 has only one susceptible epitope (Gal-2/03).

**Figure 3:**
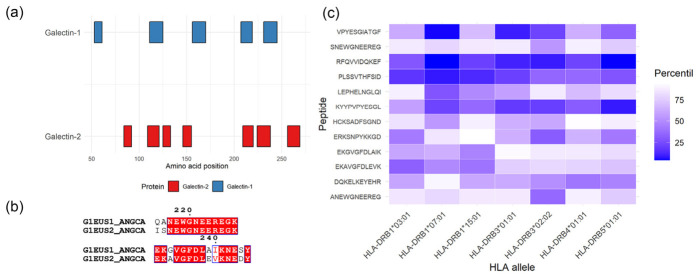
Analysis of conservation and HLA II binding of identified epitopes. Identified epitopes in *Acan-*Gal-1 and *Acan-*Gal-2 present conserved and mutated regions (**a**). Heatmap analysis of predicted HLA class II binding affinity for identified epitopes across a seven-allele panel (**b**). In C, the percentile rank values from NetMHCIIpan 4.1 are represented by a color gradient, with darker shades indicating stronger predicted binding (**c**).

*Acan-*Gal-1 and *Acan-*Gal-2 present conserved regions; for example, the epitope NEWGNEEREGK was identified in both proteins. However, mutated regions were also evident in sequence alignment; despite amino acid changes, the epitopes Gal-1/05 and Gal-2/06 were equally recognized by IgM, possibly via the conserved VGFDL region (Figure 3b). Predicted binding affinities of the identified epitopes to the seven HLA class II alleles were assessed using NetMHCIIpan 4.1. Several epitopes, including RFQVVIDQKEFRDYE and VPYESGIATGF, displayed strong binding predictions, with percentile ranks below 0.1 for multiple alleles (e.g., HLA-DRB107:01 and HLA-DRB103:01). Moderate binders, with percentile ranks between 0.5 and 2.0, were also identified, such as YPVPYESGLA for HLA-DRB3*01:01. Lower binding predictions (percentile > 3.0) were observed for epitopes like NEWGNEEREGK for some alleles. Overall, the data indicate a subset of highly promiscuous epitopes that bind multiple HLA class II molecules with high predicted affinity, suggesting their potential relevance for broad immune recognition.

### 2.4. Selection of Putative Specific Epitopes

The first goal was to eliminate cross-reactive epitopes by identifying homologous sequences of the epitopes identified for *Acan-*Gal-1 and *Acan*-Gal-2 proteins in the Pir database. The peptide search database generated from the twelve-epitope sequences identified only two epitopes (Gal-2/6 and Gal-2/7) that did not cross-react with proteins deposited in the database from several helminths (Table 1, Table S2). No unique epitopes were identified in the *Acan*-Cal-1, and seven [four (Gal-1/1, Gal-1/2, Gal-1/3, and Gal-1/4) and three (Gal-2/3, Gal-2/4, and Gal-2/5)] epitopes shared epitope sequences with *C. elegans* and *H. contortus.* However, these two parasites do not infect humans. *C. elegans* is a free-living nematode found in the soil, and *H. contortus* is a parasitic nematode of ruminants (mainly sheep and goats).

**Figure 4:**
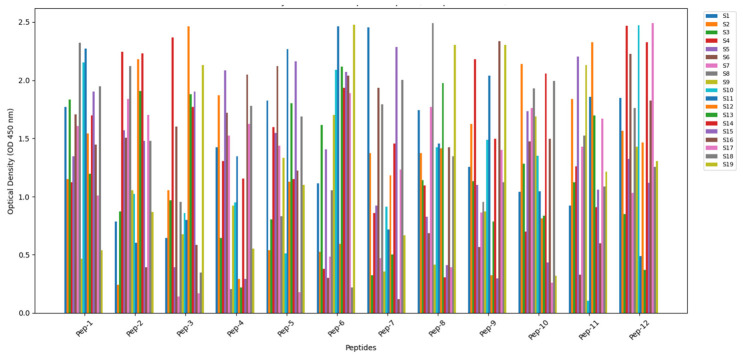
Reactivity of MAP4 synthetic peptides (Gal pep1–12) with sera from patients with Angiostrongyliasis (PS, n = 19) assessed in a pELISA. The y-axis illustrates the reactivity (positive/mean negative) of three experiments performed with sera from human-infected individuals. Each color represents the peptide indicated on the right.

Then, to confirm the immunogenicity of the epitopes, twelve MAP4 single peptides were synthesized (Table S2) and individually evaluated by ELISA assay using serum from infected patients. The results (media ± SD) are shown in Figure 4 and indicate that the epitopes have a reactivity index of 0.75 and 100% specificity and sensitivity.

**Figure 5:**
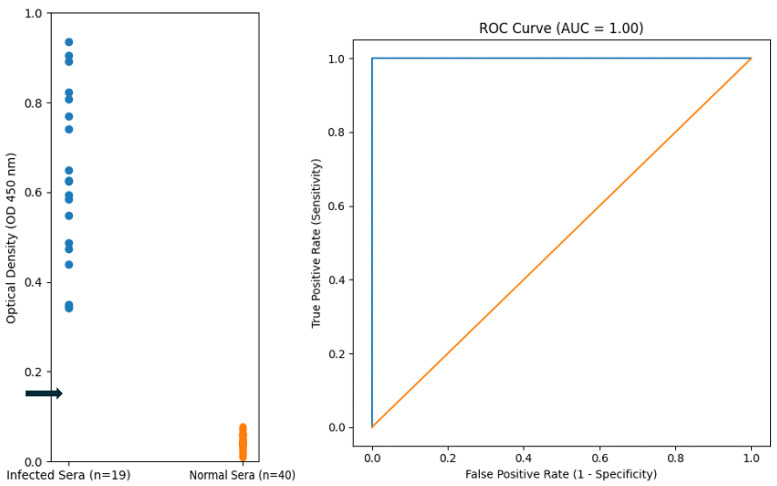
pELISA assay (**left side**) for IgM using the chimeric *A cantonensis* synthetic peptide *Acan-*CP-65 and sera from patients (PS) with angiostrongyliasis (n = 19). The data shows the reactivity index of the samples. The Kruskal–Wallis test was used to determine statistical differences, and Dunn’s multiple-comparison tests were subsequently performed. Significant differences were considered with *p* < 0.05. HS, healthy human sera (n = 40). ROC analysis (**right side**) revealed that the sensitivity and specificity of the in-house ELISA-peptide was 100% for *Acan-*CP-65. The arrow indicates the assay’s cut-off (6 × 10^−2^) detection limit.

Additionally, epitope reactivity was evaluated by ELISA (Figure 5) using a 65-mer chimeric polypeptide containing the Gal-12/6, Gal-12/7, and Gal-1/5 epitopes. The designed and synthesized polypeptide (*Acan-*CP-65) showed higher reactivity indices [*MGGS* (Gal2/06) *GPGP* (Gal2/07) *GPGP* (Gal1/5) *RSHHHHHH*]; (RI mean = 1.0 ± 0.3, positive samples = 16)] compared to the single peptides (Figure 4). This finding has potential implications for understanding immunogenicity. Notably, the sera from healthy individuals did not exhibit a significant signal.

The analytical sensitivity and ED50 (the dose required to achieve 50% of a positive signal) using the developed IgM-ELISA method were calculated to be 0.01 mg mL^−1^ and 0.052 mg mL^−1^, respectively. The intra- and inter-assay coefficients of variation (CVs) ranged from 3.31% to 11.12% and 3.99% to 8.01%, respectively.

## 3. Discussion

The diagnosis of angiostrongyliasis continues to pose significant challenges, especially during the early phase of infection, when antibody levels may remain undetectable. Current serological assays exhibit limited sensitivity and specificity, and the interpretation of Western blot results, along with the invasive nature of confirmatory procedures such as biopsies, further complicate diagnosis, particularly in endemic regions such as Brazil [37,38]. As a result, the characterization of immunodominant linear epitopes has emerged as a promising strategy for developing more accurate diagnostic tools and for improving both passive and active immunotherapeutic approaches [39,40,41].

In this study, we investigated the secreted galectin proteins Gal-1 and Gal-2 of *A. cantonensis* [42], which have been identified as having significant antigenic properties and play an important immunological role during infection [24,43]. We identified twelve IgM-reactive epitopes across these proteins (Table 1), providing further evidence that both galectins represent suitable molecular targets for diagnostic applications. These findings support their use in developing more sensitive and specific assays and offer opportunities for multiplex platforms that minimize cross-reactivity with other helminth infections.

Structural predictions revealed that most epitopes were localized exclusively within loop/coil regions, with some partially overlapping residues in α-helical segments (Table 1). This partial overlap suggests that the antigenic determinants may span boundaries between secondary structural elements. Although some segments of an epitope may reside within helices, the residues that face outward remain accessible to solvents, enabling antibodies to identify them. Thus, the structural context reveals that the identified epitopes are in accessible regions suitable for immune recognition, rather than hidden within the protein’s hydrophobic core. Complementary support for the surface exposure of these regions was obtained through hydropathy analysis. Hydropathy plots generated for both proteins also showed that the amino acid residues comprising all identified epitopes are predominantly located in hydrophilic segments of the sequence. Hydrophilic residues tend to localize on the exterior of folded proteins, where they can interact favorably with the aqueous cellular environment. Consequently, the hydropathy profiles further supported the structural predictions, indicating that the mapped epitopes are likely located on the protein surfaces.

Taken together, the integration of epitope mapping, structural modeling, and hydropathy analysis strongly supports the conclusion that the identified antigenic regions are predominantly solvent-exposed and structurally accessible. This surface localization is consistent with the typical features of B-cell epitopes and strengthens their potential relevance for applications such as serological diagnostics, antigen design, or the development of epitope-based immunoassays [44,45,46,47,48,49,50].

BLAST analyses further showed that most of these determinant sequences appear to be unique to *A. cantonensis,* strengthening their potential for diagnostic specificity. However, these results should be interpreted with caution, since sequences not deposited in the database may appear similar. Therefore, the cross-reactivity assessment was intended as a preliminary screening strategy to identify potential homologous regions in phylogenetically related organisms or host proteins that could interfere with downstream immunological assays. Only alignments involving a consecutive sequence of four amino acids were considered epitopes and potentially significant.

Importantly, this computational analysis does not directly demonstrate immunological cross-reactivity, since antibody recognition depends not only on primary amino acid similarity but also on structural accessibility, conformational context, epitope presentation, and antibody affinity. Consequently, the BLASTp-based approach was used solely to support peptide selection and to guide subsequent experimental specificity analyses using clinical sera from related infections.

Galectins are widely conserved β-galactoside-binding proteins expressed in animals, fungi, and parasites. They contain carbohydrate-recognition domains (CRDs) and are secreted via nonclassical pathways [47,48,49]. Parasite galectins share substantial homology with mammalian counterparts and have been implicated in immune modulation, inflammation, and tissue remodeling [50,51]. Although the function of galectins in *A. cantonensis* is not yet fully understood, studies in *C. elegans* indicate that upregulation of Gal-1 may protect worms against oxidative stress and enhance survival, suggesting a potential mechanism by which L5 larvae persist despite eosinophilic responses in the human central nervous system [52,53].

Previous diagnostic approaches using whole-antigen ELISAs have demonstrated inconsistent performance, and cross-reactivity with other common helminth infections remains a significant limitation [26,54]. PCR detection in cerebrospinal fluid offers high sensitivity [55,56], but the method remains expensive and inaccessible in many settings. To address these limitations, we employed peptide arrays of Gal-1 and Gal-2 and identified five antigenic determinants in Gal-1 and seven in Gal-2 recognized by patient sera. Several of these epitopes were species-specific, and ELISA assays based on MAP4 showed high sensitivity and specificity, supporting their translational potential.

Full-length *A. cantonensis* galectin 1, composed of the N-terminal CRD (residues 1–150) and the C-terminal CRD (residues 158–285), was held together via a short linker (residues 151–157). Galectin-2 has 2 domains: the NCRD (residues 1–142) and the CCRD (residues 151–278), separated by a linker (residues 143–150) [57]. Nematode galectins, much like their animal counterparts, feature highly conserved CRDs arranged in either single (prototype) or tandem-repeat formats. An example is *Toxascaris leonina* galectin (Tl-gal), a tandem-repeat galectin-9 homolog whose N- and C-terminal CRDs each adopt a β-sandwich fold composed of antiparallel β-strands that form a concave carbohydrate-binding groove [58,59]. In this structure, key motifs, HXXXR (Gal-1/3) and WGXEER (Gal-2/3), anchor the carbohydrate interaction, with critical charged residues such as Arg^61^/Arg^196^ and Glu^80^/Glu^215^ enabling binding and correct folding, and polar or aromatic residues, including His, Asn, and Trp, mediating hydrogen bonding and hydrophobic interactions with galactose-containing ligands [59]. The WGXEER (Gal-2/3) motif was a common epitope in *A. cantonensis* galectins.

Nematode galectins exhibit remarkable diversity in their domain organization and carbohydrate-binding profiles despite sharing the canonical galectin CRD fold. *C. elegans* has 11 galectins (LEC-1 to LEC-11) that conserve key β-strand motifs and residues essential for galactoside recognition, such as conserved tryptophan, arginine, and glutamate [60], but some sequence variations are responsible for differences in sugar specificity. Functional assays demonstrated that certain *C. elegans* galectins bind strongly to blood group antigens or complex N-glycans, suggesting roles in development and host–environment interactions [60]. In the nematode model *C. elegans* galectin LEC-1, which also comprises two homologous lectin domains, site-directed mutagenesis pinpointed Thr^141^ in the N-terminal domain as critical for recognizing the blood-group A hexasaccharide, illustrating how specific residues within conserved CRD frameworks modulate sugar specificity among nematode galectins [47,61].

In *A. cantonensis*, *Acan*-Gal-1 plays multiple biological and host-modulatory roles. At the L5 stage, its upregulated expression contributes to reduced fat accumulation and increased tolerance to oxidative stress, thereby supporting worm survival under inflammatory challenges [57]. Additionally, a recombinant galectin from *A. cantonensis* was successfully used in a rapid immunochromatographic test for abdominal angiostrongyliasis caused by *A. costaricensis*, demonstrating high sensitivity in detecting human antibodies and underscoring the diagnostic potential of galectins in these nematodes [62].

Though promising, these findings still have certain limitations. The relatively small sample size may not capture the genetic and immunological diversity of infected populations across different endemic regions. Moreover, since angiostrongyliasis can be caused by multiple Angiostrongylus species and strain variants, geographic and strain-specific differences may influence the breadth and applicability of the epitopes identified here. Although we have shown that they are present in all species described so far. In addition, antibody responses vary according to the timing of sample collection, and co-infections or comorbidities may influence epitope recognition patterns.

Future studies should evaluate these epitopes in larger, geographically diverse cohorts, assess their performance across different disease stages, and determine potential correlations with clinical manifestations. The development of chimeric multiepitope antigens, such as those previously explored for Mayaro virus by our group [63] and others [64,65,66], further enhances both sensitivity and specificity and represents an important direction for translational applications.

In conclusion, this study demonstrates that Gal-1 and Gal-2 contain multiple immunodominant, surface-exposed IgM epitopes with strong diagnostic potential. These findings advance peptide-based assays for angiostrongyliasis and support the development of accurate, rapid, and accessible diagnostic platforms suitable for endemic regions.

## 4. Materials and Methods

### 4.1. Patient Sera

Serum samples from 19 patients with angiostrongyliasis were obtained from the repository of the reference center of LBCE/Pontifical Catholic University of Rio Grande do Sul, Brazil (PUC-RGS) (CAAE: 46141421.9.0000.5060). Another group included 40 individuals from the blood bank donors (HEMORIO, Rio de Janeiro, Brazil). FIOCRUZ ethics committee (IOC-CAAE: 52892216.8.0000.5248 and 1.896.362).

Positive patients were included if they presented with clinical features compatible with angiostrongyliasis, particularly eosinophilic meningitis and/or neurological symptoms, together with laboratory evidence of eosinophilia in cerebrospinal fluid (CSF) and/or peripheral blood. Exclusion criteria included negative PCR results in the absence of eosinophilia; identification of alternative etiologies for meningitis or neurological symptoms (e.g., bacterial, viral, or other parasitic infections); insufficient clinical or laboratory data; and samples with inadequate quality for molecular testing.

### 4.2. Synthesis of the Cellulose-Membrane-Bound Peptide Array

A set of 285 amino acids (Gal-1) and 278 amino acids (Gal-2) overlapping pentadeca peptides, frameshifted by 5 residues, covering the primary sequence of *Acan-*Gal-1 (UniProtKB, AEK98124.1) and Gal-2 (UniProtKB, AEK98123.1) were synthesized directly onto amino-PEG500-UC540 cellulose membranes. This was achieved using the SPOT synthesis technique, as previously described [67], with an Auto-Spot Robot ASP-222 (Intavis Bioanalytical Instruments AG, Köln, Germany) and the F-moc strategy. For the immunodetection assays, the membranes were washed with TBS (50 mM Tris-buffer saline, pH 7.0) and blocked overnight with TBS-CT (Tris-buffer saline, 3% casein, 0.1% Tween 20, pH 7.0) at room temperature under agitation or overnight at 4 °C. After extensive washing with TBS-T (Tris-buffer saline, 0.1% Tween 20, pH 7.0), to remove any unbound or non-specifically bound peptides the membranes presenting the peptide libraries were incubated for 2 h with a pool of patients’ sera (1:100) in TBS-CT and then washed again with TBS-T. Antibody binding was detected using goat anti-human IgM conjugated to alkaline phosphatase (1:5000; KPL, Gaithersburg, MD, USA) for 1 h at 37 °C, followed by washing with TBS-T and CBS (50 mM citrate-buffered saline, pH 7.0). Chemiluminescent CDP-Star^®^ Substrate (0.25 mM) with Nitro-Block-II™ Enhancer (Applied Biosystems, Waltham, MA, USA) was added to complete the reaction. Negative controls (without peptide; IHLVNNESSEVIVHK (*Clostridium tetani*) precursor peptide) and positive controls (GYPKDGNA FNNLDRI-*C. tetani*–A10 and KEVPALTAVE TGATN–Poliovirus–A11) were included.

### 4.3. Scanning and Measurement of Spot Signal Intensities

The scanning and measurement of spot signal intensities involved analyzing a specified area to detect and quantify the corresponding signals. These chemiluminescent signals were detected using an Odyssey FC (LI-COR Bioscience, Lincoln, NE, USA), as previously described [67]. In brief, a digital image file was generated at 5 MP resolution, and signal intensities were quantified using TotalLab TL100 (v. 2009, Nonlinear Dynamics, Newcastle-Upon-Tyne, UK) software. The signal intensity (SI) used as a background was determined by a set of negative controls spotted on each membrane.

### 4.4. Synthesis of Peptides and Preparation of the Multi-Antigen Peptides (MAPs)

Twelve *A. cantonensis* MAPs with 4 branches (MAP4) (Table S2) were synthesized by the F-moc strategy in a synthesizer machine (MultiPep-1, CEM Corp., Charlotte, NC, USA). For the preparation of the MAPs, the classic solid-phase synthesis protocol was used, and the tetrameric F-moc-Lys2-Lys-Β-Ala Wang resin was employed, as described previously [68]. The constructs and the side chains of tetrafunctional F-moc-amino acids were protected with TFA-labile protecting groups as required. Residues corresponding to the monovalent (tail’) part of the construct, up to the first (bis-Fmoc) Lys residue initiating the dendrimer structure, were incorporated via single couplings. Once sequence assembly was completed, the F-moc groups were removed, and the peptide-resin was cleaved and fully deprotected with TFA/H2O/EDT/TIS (94/2.5/2.5/1.0 *v*/*v*, 90 min). The peptide was precipitated with chilled diethyl ether, centrifuged for 3 × 10 min at 4 °C, and the pellet was taken up in aqueous AcOH (10% *v*/*v*), dried, and stored as a lyophilized powder. When necessary, the MAPs were dissolved in water, centrifuged (10,000× *g*, 60 min, 15 °C), and the supernatant was filtered using a Centricon 10 filter. The single peptides were used without prior purification, but, when necessary, their identities were confirmed by MS (MALDI-TOF or electrospray).

### 4.5. Preparation of the Chimeric Polypeptides

For the synthesis of the single multi-epitope polypeptide (65-mer, Table S3), the epitopes were grouped into the proteins *Acan*-Gal-1 and *Acan*-Gal-2, and selected epitopes were joined with the linker GPGPG. At the amino-terminal, four additional residues (MGGS) were added, followed by a RSHHHHHH polyhistidine tail, which was added at the carboxy-terminal of the polypeptide to enable purification by immobilized metal ion affinity chromatography (IMAC).

### 4.6. ELISA-Peptide

The ELISA was carried out as described previously [67] with minor modifications. Briefly, ELISA plates (Immulon 2HB; Corning, NY, USA) were coated with 80 µL (50 µg) of each peptide prepared in coating buffer (Na_2_CO_3_–NaHCO_3_, pH 9.6) overnight at 4 °C. After each incubation step, the plates were washed three times with PBS-T (PBS with 0.1% Tween 20, adjusted to pH 7.2) and blocked (200 µL) with 2.5% BSA, and then incubated for 2 h at 37 °C. The patients’ sera were diluted (1:150) in coating buffer, and 100 µL were applied onto immunosorbent plates and incubated for 2 h at 37 °C. Following several washes with PBS-T, the plates were incubated with 100 µL goat anti-human IgM (heavy chain) conjugated to HRP (1:5000 in blocking buffer; Thermo A18835) for 2 h. The plates were developed with p-nitrophenyl phosphate (p-NPP) as a substrate. The absorbance at 405 nm was measured using a FlexStation 3 Microplate Reader (Molecular Devices, Sunnyvale, CA, USA). In all experiments, background values were subtracted from the measurement tests.

### 4.7. ELISA Validation and Performance Evaluation

The performance of the ELISA assay was evaluated by determining analytical sensitivity, ED_50_, precision (intra-assay variability), and clinical performance parameters, including sensitivity, specificity, and optimal cut-off values, using receiver operating characteristic (ROC) analysis.

Analytical sensitivity was defined as the lowest detectable concentration of the analyte that is distinguishable from the background signal. The limit of detection (LOD) was calculated as the mean optical density (OD) of blank wells plus three standard deviations (mean_blank + 3SD). Serial dilutions of the target antigen (or antibody) were tested in replicates to determine the lowest concentration consistently exceeding this threshold.

The half-maximal effective dose (ED_50_) was defined as the concentration corresponding to 50% of the maximal signal and was interpolated from the fitted curve using nonlinear regression.

Repeatability was assessed by analyzing multiple replicates (n ≥ 3) of samples within the same assay run. Intra-assay variability was expressed as the coefficient of variation (CV), calculated as CV (%) = (Standard Deviation/Mean) × 100. CV values below 10% were considered acceptable for high precision, in accordance with commonly accepted immunoassay validation criteria.

The diagnostic cut-off value was established using the mean OD of negative control samples plus 3 standard deviations.

ROC curve analysis was performed to evaluate the overall diagnostic accuracy of the assay. The area under the ROC curve (AUC) was calculated as a measure of discrimination performance, with values closer to 1.0 indicating higher diagnostic accuracy. Further, 95% confidence intervals (CIs) were estimated for AUC.

### 4.8. Structural Localization of the IgM Epitopes, Database Searches, and Computational Analysis

To ascertain the location of the epitope within the 3D molecular structure of the parasite, Galectin-1 (AF-G1EUS1-F1-v4) and Galectin-2 (AF-G1EUS2-F1-v4) were retrieved from AlphaFold 3 (https://www.ebi.ac.uk/training/online/courses/alphafold/alphafold-3-and-alphafold-server/alphafold-server-your-gateway-to-alphafold-3/, accessed on 11 February 2025). Both structures presented a high average pLDDT (>90%).

To evaluate potential cross-reactivity with other galectins or other antigenic proteins, the homology of the chosen epitopes was analyzed using the Epitope Conservancy Analysis tool available in the Immune Epitope Database (IEDB; http://tools.iedb.org/conservancy/; accessed on 25 April 2025). Additionally, epitopes were screened for HLA class II binding affinity using NetMHCIIpan 4.1 with a seven-allele panel: HLA-DRB103:01*, HLA-DRB107:01*, HLA-DRB115:01*, HLA-DRB301:01*, HLA-DRB302:02*, HLA-DRB401:01*, and HLA-DRB501:01*. Predictions were based on percentile ranks, with strong binders defined as <2% and weak binders as 2–10%, as recommended by the tool. Percentile data were compiled as a CSV file and analyzed in R (v4.x) using the ggplot2 package to generate a heatmap. A color gradient was applied, with lower percentiles (stronger predicted binding) represented by darker colors.

To curate a family of ten target sequences, Acan-Gal-1 (G1EUS1) and Acan-Gal-2 (G1EUS2) were retrieved from the UniProt database using specific criteria. Additionally, a set of sequences homologous to other proteins, identified through Blast prospection, was included after a delta-blast of the RefSeq database, with coverage greater than 80% and identity of 10–30%. From this dataset, an alignment was executed on the Mat server using blossum80 and a gap penalty of 2.5, followed by clustering to reduce the excessive number of gaps, employing the “minimum linkage” method.

Searches for possible domains and structural components characteristic of the Gal-1 and Gal-2 proteins from *A. cantonensis* were performed on the Uniprot database (http//www.uniprot. org/, accessed on 31 March 2026) based on sequence homologies with similar proteins identified in other organisms. Sequence alignments were carried out on the Clustal Omega server (http://www.ebi.ac.uk/Tools/msa/clustal, accessed on 10 July 2025). Prediction of N-linked and O-linked glycosylation was performed using the GlycoEP web server [69], which is trained on a eukaryotic glycosites database (https://webs.iiitd.edu.in/raghava/glycoep/index.html, accessed on 16 May 2025).

### 4.9. BLASTp-Based Similarity Analysis

Similarity analysis with proteins from other organisms was conducted using BLASTp version 4.0. Multiple sequence alignments were performed using the programs ClustalW (https://www.ebi.ac.uk/jdispatcher/msa/clustalo (accessed on 12 September 2025) and BioEdit (https://bioedit.software.informer.com/7.2/; accessed on 12 September 2025).

### 4.10. Statistical Analysis

All statistical analyses were performed using GraphPad Prism (version 5.0) or equivalent software. Nonlinear regression was used for curve fitting (4PL model), and ROC analysis was conducted using standard nonparametric methods. A *p*-value < 0.05 was considered statistically significant.

## 5. Conclusions

In this work, we have provided new insights, with greater coverage, into a set of human serical IgM linear B-epitopes from *A. cantonensis*. Five on *Acan-*Gal-1 and seven on *Acan*-Gal-2 are specific for the Gal proteins of Angiostrongylus. These IgM epitopes were located on the surface of the molecules and, therefore, accessible to the immune system, demonstrating the specificity and eligibility of some epitopes for entry into phase IIB studies to develop new, rapid diagnostic assays for angiostrongyliasis.

## Figures and Tables

**Table 1:** List of the identified *Acan-*Gal-1/huIgM and *Acan-*Gal-2/huIgM epitopes, structural properties, and cross-reactivity. The Gal-1 and Gal-2 *A. cantonensis* proteins include epitopes called Gal-1M and Gal-2M, which can be detected using SPOT synthesis. Following this, the epitopes were analyzed using BLASTp against a range of non-redundant sequences from multiple databases. Amino acids that line up as hits are shown in red. Cross-reactivity was identified if at least four amino acids matched up in sequence without any gaps.

Code	Epitope Sequence	Position Number	Secondary Structure ^1^	Carbohydrate Sites ^2^	Cross-Reactivity Sequence ^3^ (IEDB; Pyr Protein; UniProtKb)
Gal-2/01	SNPYKKGDD	84–92	C		*Vibrio vulnificus*
Gal-2/02	DQKELKEYEHRLP	109–121	C + S + C		Various
Gal-2/03	PLSSVTHFSIDGDVL	125–133	S + C	N-linked	*C. elegans* and *Haemonchus contortus*
Gal-2/04	YPVPYESGLA	146–155	C + S + C		*C. elegans* and *Haemonchus contortus*
Gal-2/05	S NEWGNEEREGK	209–220	C + S + C		*C. elegans* and *Haemonchus contortus*
Gal-2/06	EKAVGFDLEVKNEDY	224–238	C + S + C		None
Gal-2/07	LEPHELNGLQIGGDV	256–269	C + H + C + S + C		None
Gal-1/01	KSADFSGND	53–61	C	N-kinked	*C. elegans* and *Haemonchus contortus*
Gal-1/02	RFQVVIDQKEFRDYE	111–125	S + C + S		*Haemonchus contortus*
Gal-1/03	VPYESGIATGFQVDK	156–170	C + S + C	H*XXX*R O-linked (T) N-linked (D)	*C. elegans* and *Haemonchus contortus*
Gal-1/04	GGANEWGNEEREGKG	207–219	C + S + C	WG*X*EER	*C. elegans* and *Haemonchus contortus*
Gal-1/05	EKGVGFDLAIKNESY	231–245	C + S		*C. elegans* and *Haemonchus contortus*

^1^ Predicted by I-TASSER (accessed on 10 April 2025); ^2^ Peptide search database (UniProtKB; http://www.uniprot.org/, accessed on 31 March 2026); NQP, no query peptide (Pir Protein, accessed on 10 April 2025); ^3^ The highlighted amino acids are located within secondary structural elements rather than in coil regions. Sequences marked in gray indicate epitopes common to Gal-1 and Gal-2, and the capital letters indicate the glycosylation sites.

## Data Availability

All data are available in this paper.

## Supplementary Materials

The following supporting information can be downloaded at: https://www.mdpi.com/article/10.3390/ijms27125381/s1.
