# Supplementary material for: Immunodominant IgM Epitopes of the Angiostrongylus cantonensis Galectin-1 and Galectin-2 Proteins Recognized by Patients’ Sera: Optimization of an ELISA Assay for Human Acute Diagnosis of Angiostrongyliasis
Source: Int J Mol Sci. 2026 Jun 15;27(12):5381. doi: 10.3390/ijms27125381 (PMC13299376; doi:10.3390/ijms27125381)
Supplement: Supplementary file 1 [file ijms-27-05381-s001.zip › ijms-4265410-supplementary.pdf]

Table S1: List of the synthesized peptides (A12-C17) covering the sequence of Galectin-2 proteins from *A. cantonensis*.

Spot	Sequence	Spot	Sequence	Spot	Sequence	Spot	Sequence
A12	MAYETNYPIPYRSKL	B2	SFAKGEWGKEERKSN	B16	WGGKYYPVPYESGLA	C6	EWGNEEREGKMPFEK
A13	NYPIPYRSKLTEPFE	B3	EWGKEERKSNPYKKG	B17	YPVPYESGLAGEGLA	C7	EREGKMPFEKAVGFD
A14	YRSKLTEPFEPVQTL	B4	ERKSNPYKKGDDIDI	B18	ESGLAGEGLATGKSL	C8	MPFEKAVGFDLEVKN
A15	TEPFEPVQTLTVKGK	B5	PYKKGDDIDIRIRAH	B19	GEGLATGKSLFIYGM	C9	AVGFDLEVKNEDYAF
A16	PVQTLTVKGKTGEDS	B6	DDIDIRIRAHDSKYQ	B20	TGKSLFIYGMPEKKG	C10	LEVKNEDYAFQIMVN
A17	TVKGKTGEDSVRFTI	B7	RIRAHDSKYQIFVDQ	B21	FIYGMPEKKGKRFHI	C11	EDYAFQIMVNGERFA
A18	TGEDSVRFTINLHNA	B8	DSKYQIFVDQKELKE	B22	PEKKGKRFHINILKK	C12	QIMVNGERFASYAHR
A19	VRFTINLHNASADFS	B9	IFVDQKELKEYEHRL	B23	KRFHINILKKNGDIA	C13	GERFASYAHRLEPHE
A20	NLHNASADFSGNDVP	B10	KELKEYEHRLPLSSV	B24	NILKKNGDIALHFNP	C14	SYAHRLEPHELNGLQ
A21	SADFSGNDVPLHVSV	B11	YEHRLPLSSVTHFSI	C1	NGDIALHFNPRFDEK	C15	LEPHELNGLQIGGDV
A22	GNDVPLHVSVRFDEG	B12	PLSSVTHFSIDGDVL	C2	LHFNPRFDEKAVVRN	C16	LNGLQIGGDVEITGI
A23	LHVSVRFDEGKIVCN	B13	THFSIDGDVLITHIH	C3	RFDEKAVVRNSLISN	C17	LQIGGDVEITGIQMH
A24	RFDEGKIVCNSFAKG	B14	DGDVLITHIHWGGKY	C4	AVVRNSLISNEWGNE		
B1	KIVCNSFAKGEWGKE	B15	ITHIHWGGKYYPVPY	C5	SLISNEWGNEEREGK		

Table S2: List of the synthesized peptides (spot C18-E24) covering the sequence of Galectin-1 proteins from *A. cantonensis*.

Spot	Sequence	Spot	Sequence	Spot	Sequence	Spot	Sequence
C18	MSSPPQFLRWYEYVK	D8	DEGKMVMNTLANGEG	D22	DLYLNHVHWGGKYYP	E12	RNALQANEWGNEERE
C19	QFLRWYEYVKPVPYR	D9	VMNTLANGEGGKEER	D23	HVHWGGKYYPVPYES	E13	ANEWGNEEREGKMPF
C20	YEYVKPVPYRSLLQE	D10	ANGEGGKEERKSCPI	D24	GKYYPVPYESGIATG	E14	NEEREGKMPFEKGVG
C21	PVPYRSLLQEKIEPG	D11	GKEERKSCPIKKGDS	E1	VPYESGIATGFQVDK	E15	GKMPFEKGVGFDLAI
C22	SLLQEKIEPGQTLII	D12	KSCPIKKGDSFDIRI	E2	GIATGFQVDKTLLIF	E16	EKGVGFDLAIKNESY
C23	KIEPGQTLIIKGSTI	D13	KKGDSFDIRIRAHDD	E3	FQVDKTLLIFGTVEK	E17	FDLAIKNESYAFQIF
C24	QTLIIKGSTIDESQR	D14	FDIRIRAHDDRFQVV	E4	TLLIFGTVEKKAKRF	E18	KNESYAFQIFVNGER
D1	KGSTIDESQRFTINL	D15	RAHDDRFQVVIDQKE	E5	GTVEKKAKRFNVNLL	E19	AFQIFVNGERFTSFA
D2	DESQRFTINLHCKSA	D16	RFQVVIDQKEFRDYE	E6	KAKRFNVNLLRKNGD	E20	VNGERFTSFAHRSDP
D3	FTINLHCKSADFSGN	D17	IDQKEFRDYEHRLPL	E7	NVNLLRKNGDIALHF	E21	FTSFAHRSDPNDISG
D4	HCKSADFSGNDVPLH	D18	FRDYEHRLPLSTITH	E8	RKNGDIALHFNPRFD	E22	HRSDPNDISGLQIQG
D5	DFSGNDVPLHISVRF	D19	HRLPLSTITHLSIDG	E9	IALHFNPRFDEKAVV	E23	NDISGLQIQGDIELT
D6	DVPLHISVRFDEGKM	D20	STITHLSIDGDLYLN	E10	NPRFDEKAVVRNALQ	E24	LQIQGDIELTGIQIQ
D7	ISVRFDEGKMVMNTL	D21	LSIDGDLYLNHVHWG	E11	EKAVVRNALQANEWG		

Table S3: Synthetic peptides used in an in-house ELISA. In yellow, the extension amino acids inserted to complete the 15aa or the chimeric peptide are shown.

Epitope	Code	Sequence
Gal-2/01	Pep-1	SNPYKKGDDGPGPGG
Gal-2/02	Pep-2	DQKELKEYEHRLPGG
Gal-2/03	Pep-3	PLSSVTHFSIDGDVL
Gal-2/04	Pep-4	YPVPYESGLAGPGPG
Gal-2/05	Pep-5	SNEWGNEEREGKGPG
Gal-2/06	Pep-6	EKAVGFDLEVKNEDY
Gal-2/07	Pep-7	LEPHELNGLQIGGDV
Gal-1/01	Pep-8	KSADFSGNDGPGPGG
Gal-1/02	Pep-9	RFQVVIDQKEFRDYE
Gal-1/03	Pep-10	VPYESGIATGFQVDK
Gal-1/04	Pep-11	GGANEWGNEEREGKG
Gal-1/05	Pep-12	EKGVGFDLAIKNESY
Chimeric	*Acan*-CP-65	MGGS(Gal2/06)GPGP(Gal2/07)GPGP(Gal1/5)RSHHHHHH
